# Ginsenoside Rg1 Induces Apoptotic Cell Death in Triple-Negative Breast Cancer Cell Lines and Prevents Carcinogen-Induced Breast Tumorigenesis in Sprague Dawley Rats

**DOI:** 10.1155/2020/8886955

**Published:** 2020-10-23

**Authors:** Yan Chu, Wentao Zhang, G. Kanimozhi, G. R. Brindha, Defu Tian

**Affiliations:** ^1^Department of General Surgery, The Second People's Hospital of Yunnan Province, Kunming, Yunnan 650021, China; ^2^Department of Breast and Thyroid, Zhengzhou Central Hospital Affiliated to Zhengzhou University, Zhengzhou City, Henan Province 450000, China; ^3^Department of Biochemistry, Dharmapuram Gnanambigai Government Arts and Science College for Women, Mayiladuthurai, Tamilnadu, India; ^4^School of Computing, SASTRA Deemed University, Tirumalaisamudram, Thanjavur 613401, Tamilnadu, India; ^5^Department of General Surgery, The Fourth People's Hospital of Shaanxi, No. 512 Xianning East Road, Xi'an, Shaanxi 710043, China

## Abstract

The objective of this study is to investigate the anticancer potential of ginsenoside Rg1 using *in vitro* and *in vivo* experimental models. In this study, we found that ginsenoside Rg1 induces cytotoxicity and apoptotic cell death through reactive oxygen species (ROS) generation and alterations in mitochondrial membrane potential (MMP) in the triple-negative breast cancer cells (MDA-MB-MD-231 cell lines). We found that ginsenoside Rg1 induces the formation of gamma H2AX foci, an indication of DNA damage, and subsequent TUNEL positive apoptotic nuclei in the MDA-MB-MD-231 cell lines. Further, we found that ginsenoside Rg1 prevents 7,12-dimethylbenz (a) anthracene (DMBA; 20 mg/rat) induced mammary gland carcinogenesis in experimental rats. We observed oral administration of ginsenoside Rg1 inhibited the DMBA-mediated tumor incidence, prevented the elevation of oxidative damage markers, and restored antioxidant enzymes near to normal. Furthermore, qRT-PCR gene expression studies revealed that ginsenoside Rg1 prevents the expression of markers associated with cell proliferation and survival, modulates apoptosis markers, downregulates invasion and angiogenesis markers, and regulates the EMT markers. Therefore, the present results suggest that ginsenoside Rg1 shows significant anticancer properties against breast cancer in experimental models.

## 1. Introduction

Breast cancer is the most frequent cancer among women, impacting 2.1 million women each year, and also causes the greatest number of cancer-related deaths among women. Breast cancer is considered to be one of the genetic disorders, and it is reported as an aggressive form of cancer subtype that affects women [1]. Despite the availability of diagnostic imaging technologies and therapeutic regimes, the mammary or breast tumor remains the leading cause of mortality in women [2]. The incidence of mammary cancer is continuously increasing because of a sedentary lifestyle, environmental carcinogen exposure, and hormonal imbalance [3]. Triple-negative breast cancer (TNBC) is considered to be an aggressive form of breast cancer subtypes. These subsets of cancer patients were negative for estrogen receptors, progesterone receptors, and excess HER2 proteins. Therapeutic approaches are currently investigated by targeting DNA damage and repair mechanism. Anticancer agents induce reactive oxygen species (ROS) which subsequently induces apoptotic cell death through DNA damage mechanism. The *γ*-H2AX foci formation is an indication of double-strand DNA damage and sensitivity of cancer cells to therapy [4]. Furthermore, therapeutic agent-induced ROS cause an alteration in the mitochondrial membrane potential which results in the release of cytochrome C and induces apoptotic cell death [5]. Several studies have demonstrated that cancer cells have a unique metabolism and oxidative environment when compared to normal cells [6]. The tumor cell hypoxia environment plays a major role in cancer chemotherapy [7]. Most of the anticancer agents exploit cancer cell microenvironment, accelerate cell cycle, and alter signal transduction pathways as therapeutic targets. However, the rapidly proliferating normal cells like bone marrow cells, intestinal epithelial cells, and hair follicles were also affected by oxidative metabolism of most of the available anticancer agents [8]. Therefore, several anticancer agents are administered as prodrugs that are converted to active cytotoxic drugs upon exposure to tumor tissues exhibiting high expression of activating enzymes [9]. This targeting strategy minimizes toxicity towards normal tissues while increasing the delivery of active agent to the tumor tissue [10].

Phytochemicals possess the ability to induce apoptosis through the generation of ROS in breast cancer cells [11]. Ginsenoside Rg1 is the primary active component of Panax ginseng which has been endowed with several pharmacological properties [12]. Ginsenoside Rg1 is derived from a hydride of a dammarane and is a monomer of a tetracyclic triterpenoid derivative [13]. This molecule is mainly extracted and purified from the root or stem of ginseng. Ginsenoside Rg1 has been used to treat myocardial ischemia and atherosclerosis and used to inhibit the proliferation of vascular smooth muscle cells [14]. Ginsenoside Rg1 prevents doxorubicin-induced cardiotoxicity through the inhibition of autophagy and endoplasmic reticulum stress in mice [15]. Further, ginsenoside Rg1 promotes cerebral angiogenesis via the PI3K/AKT/mTOR signaling pathway in ischemic mice [16]. Ginsenoside Rg1 also protects H9c2 cells against nutritional stress-induced injury via aldolase/AMPK/PINK1 signaling [17]. Studies illustrate the antiproliferative effect of ginsenoside Rg1 in the cellular models [18]. Suppression of PMA-induced tumor cell invasion and migration by ginsenoside Rg1 via the inhibition of NF-kB-dependent MMP-9 expression have been reported [19]. Although similar ginsenoside effects on breast cancer cell lines were previously reported, there were no reports available on the antiproliferative effect of ginsenoside Rg1 via inducing apoptosis through the mitochondrial pathway in triple-negative breast cancer cells. Besides, ginsenoside Rg1 inhibits several carcinogenic signaling in experimental models [20]. Therefore, understanding the biochemical basis of the chemopreventive potential of ginsenoside Rg1 might attract Chinese medicine researchers. We also investigated its preventive effect in carcinogen-mediated mammary carcinogenesis in experimental models. The polycyclic aromatic hydrocarbon, 7,12-dimethylbenz[a]anthracene (DMBA), is a potent organ-specific carcinogen. Its organ-specific carcinogenicity has been well documented in several experimental models [21]. The DMBA cancer models serve as an excellent study model for experimental carcinogenesis. Systemic and local exposure of DMBA in the mammary gland generates hyperplastic alveolar nodules (HANs) and microscopic tumors [22]. Oxidative damages play a major role in the development of breast cancer. The excessive ROS formed during DMBA carcinogen exposure readily induces lipid peroxidation in the adipose tissue of mammary glands [23]. Furthermore, the carcinogen-mediated ROS activates several signaling elements involved in the multistage carcinogenesis [24]. The ROS species are reported as pre-neoplastic factors and are involved in the activation of cellular proliferation, metastatic invasiveness, and angiogenesis [25]. Human exposure to carcinogenic chemicals stimulates the development of a neoplasm in the promotion stage through influencing genetic factors related to apoptosis and cellular proliferation [26]. The activation of proto-oncogenes such as c-fos, c-jun, c-myc during mammary carcinogenesis has earlier been reported [27]. Many signal transduction pathways have been activated during chemical carcinogenesis in the mammary gland. Activation of cell survival proteins (PCNA, p21, p53, cyclin D1, and GST-P), neovascularization, and epithelial-mesenchymal transition (EMT) markers (SNAIL1 and SNAIL2) of primary mammary epithelial tumor cells leads to bloodstream intravasation during breast carcinogenesis [28, 29]. The carcinogen-induced ROS also influence the activity of proteins involved in the cell cycle, such as p53 protein [23].

Antioxidant enzymes were reported to be lowered during mammary carcinogenesis [30]. Therefore, a growing number of studies have focused on investigating the redox changes that take place in solid tumors, especially in breast cancer. The carcinogen-mediated oxidative damages and cellular signaling can be prevented by natural antioxidants [31]. Cancer chemoprevention using natural phytochemicals is considered to be a novel approach in the field of experimental carcinogenesis. Phytochemicals exhibit chemoprevention by inducing apoptosis and also modulate detoxification agents [31]. However, the chemopreventive effects of ginsenoside Rg1 in experimental mammary gland carcinogenesis have not yet been investigated. Therefore, in this study, we investigated the antiproliferative effect of ginsenoside Rg1 in triple-negative breast cancer cells and the chemopreventive effect of ginsenoside Rg1 in female Sprague Dawley rats.

## 2. Materials and Methods

### 2.1. Antiproliferative Effect of Ginsenoside Rg1 in Triple-Negative Breast Cancer Cells

#### 2.1.1. Cell Line Maintenance and Determination of Cell Viability

The triple-negative human breast carcinoma (MDA-MB-231) cells were cultured and maintained in DMEM medium supplemented with 10% fetal bovine serum and 50 units/ml penicillin-streptomycin at 37°C in a 5% CO_2_ incubator. The MTT-based cytotoxicity assay was performed to investigate the antiproliferative effect of ginsenoside Rg1 in MDA-MB-231 cells. Paclitaxel was used as the positive control for the comparison of anticancer potential of ginsenoside Rg1. The subcultured cells (1 × 10^6^ cells/well) were inoculated in 96-well plates and maintained at 37°C for 24 h. Then, the cells were treated with different concentrations (0–15 *μ*M) of ginsenoside Rg1 and further maintained for another 24 h. After the treatment period, the MTT (5 mg/ml) containing medium was added to each well. Then, the 96-well plates were maintained in a dark environment for an additional 4 h. After the removal of the MTT solution, the cells were carefully washed twice. The formed blue-colored formazan crystals were dissolved in the DMSO solution. Then, the absorbance was measured at 570 nm (Tecan, Austria).

#### 2.1.2. Analysis of Intracellular ROS

The intracellular ROS levels in MDA-MB-231 cells were analyzed using the dichlorofluoro hydrazine diacetate (DCFH-DA) method [32]. In brief, MDA-MB-231 cells (1 × 10^6^ cells/well) were treated with or without different concentrations (0–10 *μ*M) of ginsenoside Rg1 for 24 h. A PBS solution containing DCFH-DA (1 *μ*g/ml) solution was added after incubation and kept in the dark for 45 min. The fluorescence images were recorded using a fluorescence microscope at excitation of 480 nm and emission of 530 nm (Floid Cell Imaging Station, Invitrogen).

#### 2.1.3. Mitochondrial Membrane Potential (ΛΨ*m*) Assay

Mitochondrial membrane potential (ΛΨ*m*) in MDA-MB-231 cells was measured by rhodamine-123 dye [33]. MDA-MB-231 cells (5 × 10^6^ cells) were treated with different concentrations (0–10 *μ*M) of ginsenoside Rg1 for 24 h. Rhodamine-123 dye (1 *μ*g/ml) was added 2 h before the end of the experiment. The cells were washed with PBS and analyzed under a fluorescence microscope (Floid Cell Imaging Station, Invitrogen).

#### 2.1.4. Analysis of Apoptotic Morphological Changes

Acridine orange/ethidium bromide (AO/EtBr) dual staining was performed to assess apoptotic changes after the treatment with ginsenoside Rg1 (0–10 *μ*M). Acridine orange enters in the viable and dead cells, whereas the ethidium bromide enters only into the dead cells. In brief, control and ginsenoside Rg1 treated MDA-MB-231 cells were stained with a 1 : 1 ratio of acridine orange and ethidium bromide for 10 min. The stained cells were then examined under the fluorescence microscope in both red and green channels (Floid Cell Imaging Station, Invitrogen).

#### 2.1.5. Estimation of DNA Damage by *γ*H2AX Foci Assay

After ginsenoside Rg1 treatment for 24 h, the breast cancer cells were fixed in the ice-cold microscopic slide using 50% methanol and 50% ethanol for 20 minutes at room temperature. Then, the treated cells were permeabilized with 0.5% Triton X-100 and then blocked with 0.2% skimmed milk. Cells were then stained with anti-*γ*-H2AX antibody (dilution of 1 : 800 in 1% BSA) and anti-mouse FITC-secondary antibody. Coverslips were mounted with mounting medium containing DAPI to counterstain cellular nuclei. Images were collected using a fluorescence microscope. Cells were analyzed by confocal microscopy to visualize the *γ*H2AX foci formation.

#### 2.1.6. Terminal Deoxynucleotidyl Transferase-Mediated dUTP Nick-End Labeling (TUNEL) Assay

After treatment with different concentrations (0–10 *μ*M) of ginsenoside Rg1 for 24 h, the cells were harvested and washed with PBS. DNA strand breaks in MDA-MB-231 cells were detected by the APO-BrdU TUNEL assay kit (Invitrogen) as per the manufacturer's instructions. Samples were analyzed with fluorescent microscopy (Floid Cell Imaging Station, Invitrogen).

### 2.2. Chemopreventive Effect of Ginsenoside Rg1 in DMBA-Induced Mammary Carcinogenesis

#### 2.2.1. Experimental Animals

Six-to-seven-week-old female Sprague Dawley rats (weighing 120–130 g) were purchased and used for the experimental carcinogenesis. We conducted the experiments as per the IAEC approval of the Second People's Hospital of Yunnan Province (YNSDERMYY0921). The experimental animals were acclimatized to the control diet and laboratory environment for 1 week period. The animals were maintained under controlled conditions of temperature (24 ± 2°C), humidity (50 ± 10%), and 12 h light/dark cycle. Feed and water were provided *ad libitum*.

#### 2.2.2. Experimental Plan

The experimental animals were divided into four groups, and each group comprised of six animals (*N* = 6), and in each group, six samples were processed (*n* = 6). The experimental dose of ginsenoside Rg1 was determined by acute toxicity study (Supplementary Table 1). Group I rats served as an untreated control. Group II rats received oral administration of ginsenoside Rg1 at a dose of 10 mg/kg bw (5 mL/kg bw) alone throughout the experimental period. Groups III rats received a single subcutaneous injection of DMBA (20 mg/rat) near the mammary gland, at the end of the first week. Group IV rats were administered ginsenoside Rg1 orally at a dose of 10 mg/kg bw plus DMBA (20 mg/rat). At the end of the 16th week, rats were fasted overnight and sacrificed by cervical decapitation. Blood samples were collected in heparinized tubes, and the separated plasma was used for the biochemical analysis. Mammary tissue was excised immediately from the rats and stored in ice-cold containers. Tissues were homogenized with a suitable buffer and centrifuged at 3000*g*, and the supernatant was used for biochemical estimations on the same day of sacrifice.

#### 2.2.3. Analysis of Oxidative Damage-Related Parameters

Mammary tissue was rinsed in ice-cold saline. A known amount of the tissue was homogenized in 0.1 M Tris-HCl buffer (pH 7.4), at 4°C, in a Potter-Elvehjem homogenizer with a Teflon pestle at 600 g for 3 min. The homogenate was centrifuged at 3000*g* for 10 min at 4°C. The supernatant was collected as tissue homogenate, which was used to assay various biochemical parameters. The concentration of mammary tissue TBARS was estimated by the method of [34]. The concentration of mammary tissue LOOH was estimated by the method of [35]. The SOD, catalase, and glutathione peroxidase activities in mammary tissues were determined by the method of [36]. GSH levels were determined by the method of [37].

#### 2.2.4. Analysis of Carcinogenesis-Related Gene Expression by a qRT-PCR Array

The total RNA content was isolated from control, DMBA control, and DMBA plus ginsenoside Rg1 treated mammary tissues using an RNeasy mini kit and used for qRT-PCR arrays. The purity of the isolated RNA was measured by Nanodrop spectrophotometer. The cDNA was reverse transcribed using a First Strand cDNA Synthesis Kit and used for PCR amplification by SYBR green chemistry using the Qiagen kit. The relative expression pattern of cell survival genes and cell cycle regulators (TP53, BCl2, and CDK2), growth factors (TGFB1, VEGFA, and EGFR), transcription factors involved in mammary carcinogenesis (NF-*κ*B and STAT-3), proangiogenic molecules (RECK, MMP-2, MMP-9, TIMP-1, HIF-1*α*, VEGF, and VEGFR2), neovascularization and inflammation (MAPK, iNOS, MMP2, and MMP9), epithelial-mesenchymal transition (SNAIL2), and proto-oncogenes (MYC and HER2) was analyzed by a qRT-PCR array. The fold changes of gene expression were plotted as cluster grams.

#### 2.2.5. Statistical Analysis

Statistical analysis was performed using SPSS 16 (SPSS, Inc., Chicago) statistical package. The data are expressed as mean ± standard deviation (SD). One-way analysis of variance (ANOVA) followed by Duncan multiple range test (DMRT) comparison method was used to correlate the difference between the variables. Data are considered statistically significant if *p* values are less than 0.05.

## 3. Results

### 3.1. Antiproliferative Effect of Ginsenoside Rg1 in Triple-Negative Breast Cancer Cells

#### 3.1.1. Cytotoxicity of Ginsenoside Rg1 in MDA-MB-231 Cells

**Figure 1:**
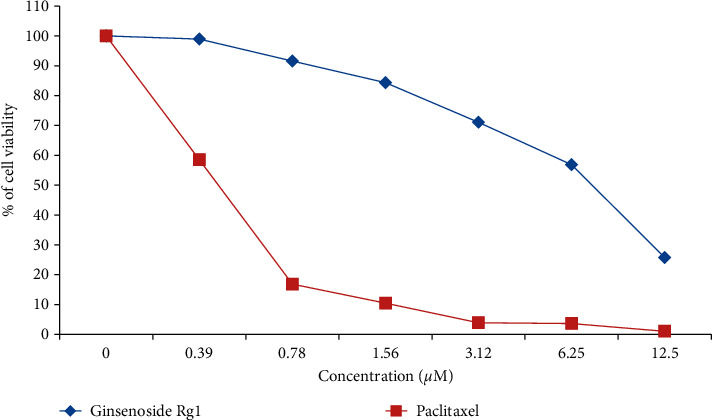
Antiproliferative activity of ginsenoside Rg1 in triple-negative breast cancer cells. MTT assay was performed, and after 72 h, the cytotoxicity was measured and compared with standard anticancer drug paclitaxel. Ginsenoside showed IC_50_ value of 8.12 *μ*M and paclitaxel showed IC_50_ value of 0.48 *μ*M in MDA-MB-231 cell lines.

The present study investigated the cytotoxic potential of ginsenoside Rg1 in MDA-MB-231 cells. A significant reduction in cell viability was found with different concentrations of ginsenoside Rg1 treatment in MDA-MB-231 cells (IC_50_ = 8.1 *μ*M). The pattern of cytotoxicity was comparable to the standard positive control paclitaxel; however, paclitaxel shows greater cytotoxicity than ginsenoside Rg1 (IC_50_ = 0.48 *μ*M). Further, the cell viability was found to be decreased in a concentration-dependent manner when compared with untreated control MDA-MB-231 cells. The decrease in % cell viability was found higher in 12.5 *μ*M of ginsenoside Rg1 treatment in MDA-MB-231 cells. The standard positive control paclitaxel showed IC_50_ values. The results confirm that CSME exerts a significant cytotoxic effect in MDA-MB-231 cells (Figure 1).

#### 3.1.2. Ginsenoside Rg1 Generates ROS-Induced Apoptosis in MDA-MB-231 Cells

**Figure 2:**
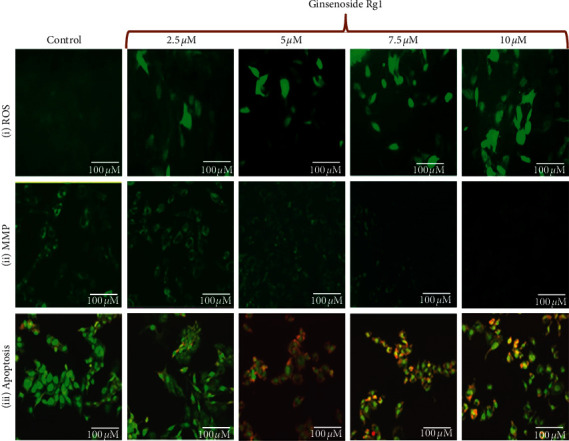
Effect of ginsenoside Rg1 on (i) ROS generation, (ii) mitochondrial membrane potential alteration, and (iii) apoptotic morphological changes by fluorescence microscopy.

The results from fluorescence microscopic examination clearly showed the formation of intracellular ROS in CSME treated MDA-MB-231 cells (Figure 2(i)). Our results show that ginsenoside Rg1 induced intracellular ROS in a concentration-dependent manner in MDA-MB-231 cells. The levels of intracellular ROS (fluorescence intensity) observed with 7.5 *μ*M and 10 *μ*M of ginsenoside Rg1 treatment were found to be high when compared to other concentrations. No significant ROS levels were observed in untreated MDA-MB-231 cells.

Further, we also observed a considerable loss of mitochondrial membrane potential (ΛΨ*m*) after ginsenoside Rg1 treatment in MDA-MB-231 cells. The loss of ΛΨ*m* followed a concentration-dependent manner. The loss of ΛΨ*m* was observed to be high with 10 *μ*M of ginsenoside Rg1 treatment in MDA-MB-231 cells. The results suggest that ginsenoside Rg1 induces redox instability which leads to the loss of ΛΨ*m* in MDA-MB-231 cells (Figure 2(ii)).

**Figure 3:**
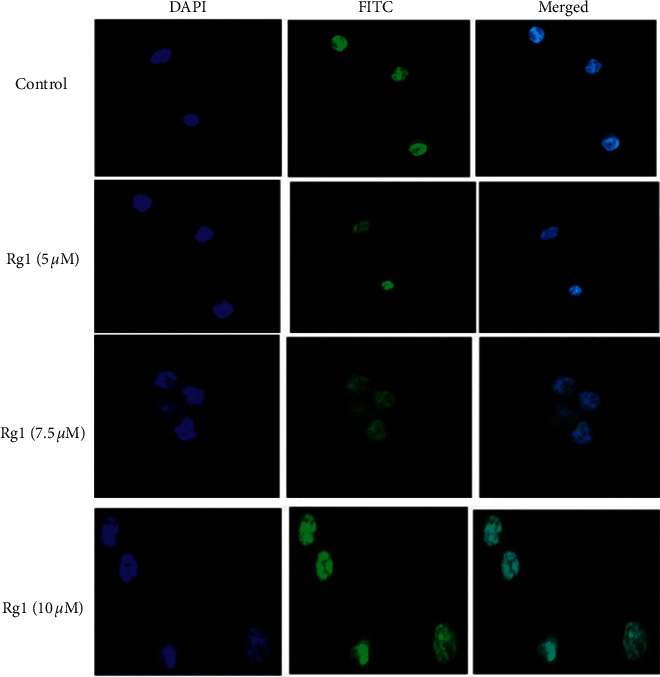
Effect of ginsenoside Rg1 on gamma H2AX loci formation in MDA-MB-231 cells. The cells were treated with different concentrations of Rg1 for 24 h and then analyzed for foci formation by immunocytochemistry. The binding of mAb gH2A was revealed with FITC-labeled goat anti-mouse immunoglobulins (green). The nuclei were counterstained with DAPI (blue).

We observed that ginsenoside Rg1 treatment was able to induce initial events of apoptosis (yellowish-orange chromatin) with some cells undergoing cell death (red chromatin) (Figure 3). The ginsenoside Rg1 treatments induced significant apoptotic morphological alterations in MDA-MB-231 cells. The apoptotic effects of ginsenoside Rg1 in MDA-MB-231 cells were noticed to follow a concentration-dependent manner. Higher levels of apoptosis were observed with 10 *μ*M of ginsenoside Rg1 treatment in MDA-MB-231 cells (Figure 2(iii)).

#### 3.1.3. Ginsenoside Rg1 Induces *γ*H2AX Foci Formation in MDA-MB-231 Cells

The immunofluorescence study showed that ginsenoside Rg1 treatment was able to induce considerable DNA damage in MDA-MB-231 cells. The *γ*H2AX foci formation as a function of DNA damage in MDA-MB-231 cells with ginsenoside Rg1 followed a concentration-dependent manner. Higher levels of *γ*H2AX foci formation were observed with 10 *μ*M of ginsenoside Rg1 treatment in MDA-MB-231 cells (Figure 3)

#### 3.1.4. Ginsenoside Rg1 Induces TUNEL Positive Apoptotic MDA-MB-231 Cells

**Figure 4:**
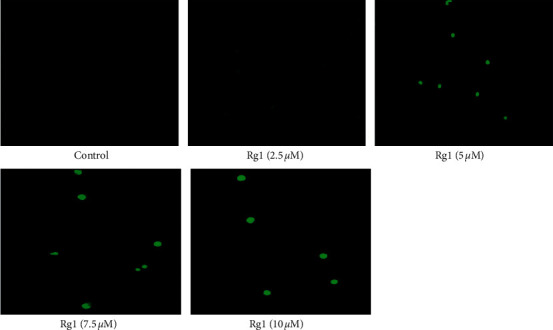
Effect of ginsenoside Rg1 on DNA fragmentation by TUNEL assay. TUNEL-positive lesions are enumerated as TUNEL-positive nuclei in MDA-MB-231 cells. The images were taken under a fluorescence microscope (Floid Cell Imaging Station, Invitrogen). Representative results of three independent experiments are shown for each experiment.

In the present study, we observed a considerable level of apoptosis in MDA-MB-231 cells treated with different concentrations (0–10 *μ*M) of ginsenoside Rg1 when compared to untreated control cells. The ginsenoside Rg1 treatment induced significant dUTP-FITC-positive cells as a result of significant DNA fragmentation in MDA-MB-231 cells (Figure 4). All these results prove that ginsenoside Rg1 exhibits a considerable potential to induce apoptotic events in MDA-MB-231 cells.

### 3.2. Chemopreventive Effect of Ginsenoside Rg1 in Experimental Animals

#### 3.2.1. Effect of Ginsenoside Rg1 on Tumor Incidence in DMBA Experimental Rats

This study demonstrated that 100% tumor incidence was observed in DMBA treated rats. Oral administration of ginsenoside Rg1 at a dose of 10 mg/kg bw significantly (*p* < 0.05) prevented the tumor incidence, the total number of tumors, tumor volume, and tumor burden when compared to DMBA alone treated groups (Table 1).

#### 3.2.2. Effect of Ginsenoside Rg1 on Antioxidant Status

Table 2 shows the status of mammary tissue enzymatic and nonenzymatic antioxidants, respectively, in control and experimental rats. The activities of SOD, CAT, and GPx and the levels of GSH were significantly (*p* < 0.05) decreased in DMBA alone treated rats when compared with the control group. Oral administration of ginsenoside Rg1 (10 mg/kg bw) significantly (*p* < 0.05) restored the activities of SOD, CAT, and GPx and levels of GSH when compared with DMBA alone treated rats. However, there was no significant difference (*p* < 0.05) in the rats administered with ginsenoside Rg1 alone.

#### 3.2.3. Effect of Ginsenoside Rg1 on Lipid Peroxidation Status

Table 3 shows the status of lipid peroxidation in mammary tissues of control and experimental rats. The levels of TBARS and LOOH were significantly (*p* < 0.05) increased in DMBA alone treated rats when compared to the control rats. Oral administration of ginsenoside Rg1 decreases the levels of TBARS and LOOH when compared with DMBA alone treated rats (*p* < 0.05). However, there was no significant difference in the rats administered with ginsenoside Rg1 alone.

#### 3.2.4. Analysis of Carcinogenesis-Related Gene Expression by PCR Array

**Figure 5:**
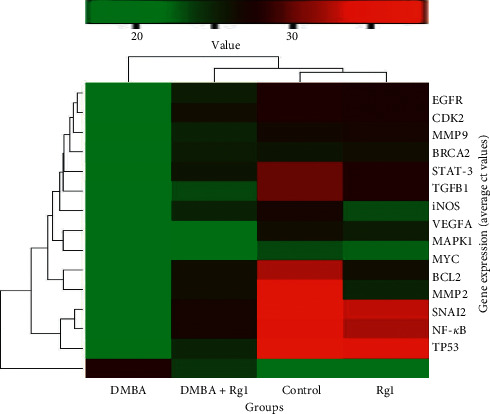
Expression levels of carcinogenesis-linked gene expression in the DMBA and DMBA plus ginsenoside Rg1 treated breast cancer tissue. The mRNA expression pattern of genes involved in cell proliferation, apoptosis, inflammation, and angiogenesis and EMT-linked genes was analyzed. The total mRNA was isolated from fresh breast tumor tissues and was detected using a custom qRT-PCR array following the manufacturer's instructions. The cluster gram results of three independent experiments were analyzed using the SA Biosciences online tool.

The relative expression pattern of cell survival genes (TP53, BCl2, and CDK2), transcription factors involved in mammary carcinogenesis (NF-*κ*B and STAT-3), proangiogenic molecules (MAPK, iNOS, MMP2, and MMP9), and growth factors (TGFB1, VEGFA, and EGFR) was found to be overexpressed in the mammary tumor tissue homogenate. Furthermore, epithelial-mesenchymal transition (SNAIL2) and proto-oncogenes (MYC and HER2) were found to be upregulated in the DMBA-induced breast cancer tissues. Conversely, the ginsenoside Rg1 treatment prevented the overexpression of carcinogenesis, angiogenesis, and EMT-related gene expression towards a normal level (Figure 5).

## 4. Discussion

The growing incidence of cancer patients and the limitations of synthetic chemotherapy due to their side effects have always been a major concern to the cancer scientists. Ginseng has been traditionally used for the prevention and treatment of various chronic diseases [38, 39]. In the acidic tumor microenvironment, most of the antioxidant phytochemicals behave as prooxidants and generate reactive oxygen species [40]. This ROS subsequently alters mitochondrial membrane potential and induces apoptotic cell death of cancer cells [41]. In this study, we tested the cytotoxicity of ginsenoside Rg1 in MDA-MB-231 cells. The IC_50_ value of ginsenoside Rg1 in MDA-MB-231 cells was found to be 8.12 *μ*M. Several studies showed the cytotoxicity and anticancer properties of ginsenosides, including induction of apoptosis, in several typical cancer types, such as lung, breast, and colorectal cancer cells as well as neuroblastoma cells [20, 42]. The formation of intracellular ROS is a hallmark of oxidative stress [43]. Ginsenoside Rg1 treatment generates ROS, alters mitochondrial membrane potential, and subsequently induces apoptotic cell death in MDA-MB-231 cells. The alteration of MMP has been considered as the initial event of apoptotic cell death in cancer cells. Several ginsenosides induce apoptosis and paraptosis by activating both p53 and NF-kB through ROS generation [44]. A prominent *γ*H2AX foci formation was in ginsenoside Rg1 treated breast cancer cells in a concentration-dependent manner. The ROS generation during acidic cancer cell environment by ginsenoside Rg1 might cause oxidative DNA damages and subsequent double-strand DNA breaks. The foci observed after 24 h of Rg1 treatment indicate the formed DNA damage leading to apoptosis. Similarly, ellagic acid, a phenolic phytochemical, enhances the apoptotic sensitivity of breast cancer cells through the formation of *γ*H2AX foci [45]. The formation of apoptotic cell death and DNA fragmentation during ginsenoside Rg1 has further been confirmed by the results of TUNEL positive cell formation. Collectively, these data suggest than ginsenoside Rg1 induces apoptotic cell death through the formation of ROS and subsequent DNA damage in breast cancer cells. Similarly, ginsenoside compound K induces ROS mediated apoptosis in neuroblastoma cell lines [46].

Ginsenosides have been extensively investigated and emphasized in cancer chemoprevention and therapeutics. Further, ginsenosides were found to protect human cells by removing hydrogen peroxide and hydroxyl radicals [47]. Ginsenosides possess higher antioxidation activity and stronger anticancer activity [48]. Previous results showed the structure and antioxidant relationship of ginsenoside Rg1 and illustrated the potential of ginsenosides for the treatment of diseases associated with free radicals [49]. Ginsenoside Rg1 possesses multiple hydroxyl groups in its structure that typically exhibit antioxidant and chemopreventive agents. The oxidative stress can be considered as the interplay between antioxidant and prooxidant status of the cellular system. As ginsenoside Rg1 exhibits excellent antioxidant properties in this study, it could prevent reactive oxygen species-mediated carcinogenesis. In this study, ginsenoside Rg1 prevented DMBA-induced mammary carcinogenesis in the rats. The DMBA has been listed as group I carcinogen, and it induces the generation of reactive oxygen species. This ROS generation has been associated with the depletion of the endogenous antioxidant which is the most prominent event in the development of carcinogenesis. We found a severe oxidative tumor environment in the DMBA treated rats. The results also illustrated that there was an increased tumor incidence and tumor burden in the DMBA treated animals. Conversely, ginsenoside Rg1 pretreatment prevented the DMBA-mediated tumor incidence and tumor volume. A recent study showed that ginsenosides regulate tumor microenvironment via suppressing tumor angiogenesis-related pathways [50]. The DMBA has been reported to generate many reactive intermediates (O_2_^−^, H_2_O_2_, and OH^−^) that potentiate oxidative stress primarily by binding to nucleophilic sites of cellular macromolecules resulting in adenocarcinoma in the mammary gland [39]. We found elevated TBARS and LOOH as a result of free radical-mediated lipid peroxidation in DMBA rats. Excessive lipid peroxides in serum and tissues can be correlated with the proliferation and metastasis of breast cancer cells [51]. The antilipid peroxidative effect of ginsenoside Rb1 and Rg1 has well been documented [52]. The Rb1, at a dose of 50 and 25 mg/kg/day × 3 ip, inhibited MDA formation in the liver homogenate of rats by 26.8%. Similarly, ginsenoside Rg1 protects against liver injury by ameliorating lipid peroxidation, endoplasmic reticulum stress, and inflammasome activation [53]. Elevated levels of free radicals and lipid peroxidation are important mediators of carcinogenesis. The antioxidant phytochemicals scavenge DMBA-induced free radicals, thereby effectively preventing lipid peroxidation. The attenuated formation of TBARS and LOOG during ginsenoside Rg1 treatment could directly be correlated to its antioxidative property. Ginsenoside Rg1 transfers hydrogen atom or electron to neutralize the free radical, thereby preventing chain reactions and behaving as antioxidant in normal cells. In the cancer cell acidic environment, ginsenoside Rg1 was found to be unable to donate electrons. Further, this antioxidant action is mediated through free radical scavenging and through upregulation of antioxidant enzyme [49].

Antioxidant systems are critical in protecting against tumor-promoting agents. Interestingly, carcinogenesis is often accompanied by a decrease in the activity of cellular endogenous antioxidant enzymes. The antioxidant enzymes SOD, CAT, GPx, and nonenzymatic antioxidant GSH limit the effects of oxidants against oxidative cell injury [54]. These enzymes work synergistically to eliminate active oxygen species which are involved in oxidative damage [55]. The decreased activities of these enzymic antioxidants observed in DMBA-induced rats might be due to severe increased oxidative. The reduced GSH is an antioxidant present in high amounts, especially in the mammary tissue, and protects the cellular milieu against oxidative damage. The present results show that ginsenoside Rg1 treatment protects the DMBA-mediated depletion in GSH levels in mammary tissue. The low-level activities of antioxidant enzymes like SOD, CAT, and GPx in DMBA-treated mice show the poor antioxidant status in mammary tissue. Researchers showed the attenuated activities of SOD, CAT, and GPx in mammary carcinomas [56]. The increase in antioxidant enzymes by ginsenoside Rg1 reflects that it inhibits the process of oxidative stress-induced carcinogenesis. Several reports suggest that GSH and enzymatic antioxidants are more important for the prevention of carcinogenesis [57].

Identification of potential molecular targets to prevent cancer initiation and progression by natural products gains growing interest among cancer researchers. Cell growth and proliferation are coordinated with cell cycle checkpoints and apoptosis [58]. We observed overexpression of prominent proliferation markers (Figure 5) during DMBA-mediated carcinogenesis. The overexpression of BCL2 and CDK2 and downregulation of TP53 have been observed in DMBA-treated mammary tumors. Conversely, these apoptotic molecules were modulated by Rg1 treatment. There were several transcription factors involved in DMBA-mediated mammary carcinogenesis [59]. The present results illustrate that Rg1 treatment prevented the activation of proinflammatory and carcinogenic transcription factors like NF-*κ*B and STAT-3 in mammary tissue. The modulatory potential of ginsenoside Rg1 against NF-kB mediated apoptosis and inflammation in the experimental models [60]. Further, ginsenoside Rb1 exerts anti-inflammatory effects *in vitro* and *in vivo* models by modulating toll-like receptor 4 dimerization and NF-kB/MAPKs signaling pathways [61]. Furthermore, ginsenoside Rg1 was found to be effective in regulating PXR/NF-kB signaling to attenuate dextran sulfate sodium-induced colitis [62]. Our cluster gram results illustrate that ginsenoside Rg1 prevents DMBA-mediated overexpression of proangiogenic molecules and growth factors in the mammary tissue. The negative regulation of proinflammatory marker (TNF-*α*, IL-1*β*, and IL-6) and angiogenic marker expression was recently found for ginsenoside Rg1 in M1-polarized macrophages and microglia [63]. Our results also showed that ginsenoside Rg1 treatment prevented the overexpression of EMT factor SNAIL2 in the mammary tissue. Similar to our findings, ginsenosides suppress EMT activation to attenuate fibrosis. Our results also showed that ginsenoside Rg1 treatment prevented the DMBA-mediated activation of proto-oncogenes such as MYC and HER2 in the mammary tissue. Ginsenoside Rg1 has been reported to inhibit oncogenes such as c-myc and c-fos and downregulate nucleophosmin [49, 64]. Therefore, our present findings are consistent with the antiproliferative and chemopreventive effects of ginsenoside Rg1 documented in other malignant cell lines. It was also shown that oxidation of ginsenoside Rg1 possibly leads to the formation of prooxidants. This prooxidant action of ginsenoside may be an important mechanism of their anticancer properties. It is found that the central structures of ginsenosides, either protopanaxadiol or protopanaxatriol, play major role in prooxidative mechanism [65].

Published clinical studies suggested that ginsenosides were a good antitumor agent by improving the immune function and the quality of life of cancer patients [66]. Pharmacokinetics of single and multiple doses (10–60 mg) of similar anticancer ginsenosides was found to be well tolerated in healthy Chinese volunteers [67]. Therefore, dose used for chemopreventive potential of ginsenoside Rg1 in the experimental rats could be translated to the human. However, its pharmacokinetics, toxicity profile, and safety need to be assessed.

## 5. Conclusion

Thus, ginsenoside Rg1 acts as a potent antiproliferative and chemopreventive agent against breast cancer. We found that ginsenoside Rg1 induced apoptotic cell death through ROS generation in triple-negative breast cancer cell lines. Besides the formation of *γ*H2AX foci, ginsenoside Rg1-induced ROS alters the mitochondrial membrane potential in the breast cancer cells. Further, oral administration of ginsenoside Rg1 inhibited the DMBA-mediated tumor incidence and prevented the elevation of oxidative damage markers in experimental rats. Further, the ginsenoside Rg1 treatment inhibits the development of DMBA-induced mammary carcinomas through modulating molecules involved in cell proliferation, apoptosis, invasion, and angiogenesis as well as EMT transition. Hence, ginsenoside Rg1 may be considered as a potential chemopreventive agent.

## Figures and Tables

**Table 1:** Effect of ginsenoside Rg1 on tumor incidence, the total number of tumors, and tumor volume of control and experimental rats.

Experimental groups	Tumor incidence (%)	Number of tumors (*n*)	Tumor volume (mm^3^/animal)
Sham control	0	0/8	0
Ginsenoside Rg1	0	0/8	0
DMBA	100	8/8	20.85 ± 2.94
DMBA plus ginsenoside Rg1	50	4/8	10.31 ± 1.98

Tumor volume was measured using the formula *V* = 4/3*π* (D1/2) (D2/2) (D3/2), where D1, D2, and D3 are the three diameters mm of the tumor; mm indicates total number of rats bearing tumors.

**Table 2:** Effect of ginsenoside Rg1 on antioxidant status in plasma of control and experimental rats.

Experimental groups	SOD^*∗*^	Catalase^*∗∗*^	GPx^*∗∗∗*^	GSH
Sham control	15.70 ± 1.73^a^	56.00 ± 4.26^a^	13.75 ± 1.05^a^	15.55 ± 1.45^a^
Ginsenoside Rg1	14.10 ± 1.48^a^	53.02 ± 4.05^a^	13.82 ± 1.16^a^	13.40 ± 1.02^a^
DMBA	5.95 ± 1.43^c^	26.66 ± 4.50^c^	6.88 ± 0.67^c^	6.00 ± 0.45^c^
DMBA plus ginsenoside Rg1	10.87 ± 0.90^b^	45.02 ± 3.44^b^	10.89 ± 0.83^b^	12.00 ± 0.91^b^

*U* ^*∗*^: the amount of enzyme inhibiting 50% NBT reduction/min. *U*^*∗∗*^: *μ*mol of hydrogen peroxide consumed/min. *U*^*∗∗∗*^: *μ*g of reduced glutathione consumed/min. Values are expressed as mean ± SD for eight rats in each group. Values not sharing a common superscript differ significantly at *p* < 0.05 (DMRT).

**Table 3:** Effect of ginsenoside Rg1 on lipid peroxidation by-product status in plasma and mammary tissues of control and experimental rats.

Experimental groups	Plasma levels		Mammary tumor	
	TBARS (mm/dL)	Lipid hydro peroxides (mm/dL)	TBARS (mm/100 g tissue)	Lipid hydroperoxide (mm/100 g tissue)
Sham control	2.83 ± 0.71^a^	2.13 ± 0.56^a^	4.05 ± 0.88^a^	3.11 ± 0.28^a^
Ginsenoside Rg1	3.06 ± 0.79^a^	1.90 ± 0.74^a^	4.45 ± 1.03^a^	2.51 ± 0.75^a^
DMBA	6.31 ± 1.03^c^	7.64 ± 1.00^c^	11.13 ± 0.79^c^	6.75 ± 1.00^c^
DMBA plus ginsenoside Rg1	4.13 ± 0.75^b^	4.11 ± 1.53^b^	6.83 ± 0.56^b^	3.12 ± 1.53^b^

Values are expressed as mean ± SD; *n* = 8. Values not sharing a common superscript (a, b, c, etc.) significantly differ at *p* < 0.05 (DMRT).

## Data Availability

The underlying data are available from the corresponding author.

## Supplementary Materials

Supplementary Materials Supplementary Table 1: effect of various concentrations (2.5–1280 mg/kg b.wt) of ginsenoside Rg1 on the survival of Swiss albino mice. Different doses of ginsenoside Rg1 were administered to mice and mortality was observed for 14 consecutive days.
