# Supplementary material for: Ginsenoside Rg1 Induces Apoptotic Cell Death in Triple-Negative Breast Cancer Cell Lines and Prevents Carcinogen-Induced Breast Tumorigenesis in Sprague Dawley Rats
Source: Evid Based Complement Alternat Med. 2020 Oct 23;2020:8886955. doi: 10.1155/2020/8886955 (PMC7607905; doi:10.1155/2020/8886955)
Supplement: Supplementary Materials — Supplementary Table 1: effect of various concentrations (2.5–1280 mg/kg b.wt) of ginsenoside Rg1 on the survival of Swiss albino mice. Different doses of ginsenoside Rg1 were administered to mice and mortality was observed for 14 consecutive days. [file 8886955.f1.docx]

***Acute toxicity of Ginsenoside Rg-1***

The administration of different concentration of ginsenoside Rg-1 (2.5-1280 mg/kg b.wt) has not induced mortality during the 14 days observation period. However, 16.6% animals died when the ginsenoside Rg-1 dose was raised to 20 – 160 mg/kg b.wt. A further increase in the dose ginsenoside Rg-1 (320 mg/kg.b.wt.) resulted in 33.3% mortality. About 50% reduction in the survival of mice was observed 640 mg/kg.b.wt. Finally, 1280 mg/kg.b.wt. has been observed 66.66% mortality in treatment.

| **Mortality on different day post drug treatment** | | | | | | | | | | | | | | | | |
| --- | --- | --- | --- | --- | --- | --- | --- | --- | --- | --- | --- | --- | --- | --- | --- | --- |
| **Ginsenoside Rg-1 (mg/kg b.wt)** | **1** | **2** | **3** | **4** | **5** | **6** | **7** | **8** | **9** | **10** | **11** | **12** | **13** | **14** | **% Mortality** | **Survivors/ Total** |
| 2.5 |  |  |  |  |  |  |  |  |  |  |  |  |  |  | 0 | 6/6 |
| 5.0 |  |  |  |  |  |  |  |  |  |  |  |  |  |  | 0 | 6/6 |
| 7.5 |  |  |  |  |  |  |  |  |  |  |  |  |  |  | 0 | 6/6 |
| 10 |  |  |  |  |  |  |  |  |  |  |  |  |  |  | 0 | 6/6 |
| 20 |  |  |  |  |  |  |  |  |  |  |  |  | 1 |  | 16.6 | 1/6 |
| 40 |  |  |  |  |  |  |  |  |  |  | 1 |  |  |  | 16.6 | 1/6 |
| 80 |  |  |  |  |  |  | 1 |  |  |  |  |  |  |  | 16.6 | 1/6 |
| 160 |  |  |  |  | 1 |  |  |  |  |  |  |  |  |  | 16.6 | 1/6 |
| 320 |  |  |  | 1 |  |  | 1 |  |  |  |  |  |  |  | 33.3 | 2/6 |
| 640 |  |  | 1 | 1 |  |  |  | 1 |  |  |  |  |  |  | 50 | 3/6 |
| 1280 |  |  | 1 | 1 | 1 |  |  |  |  |  | 1 |  |  |  | 66.66 | 4/6 |

Table 1. Effect of various concentrations (2.5-1280 mg/kg b.wt) of ginsenoside Rg-1 on the survival of non-irradiated Swiss albino mice. Different doses of ginsenoside Rg-1 were administered to mice and mortality for 14 consecutive days.
